# Influence of Protein – Micelle Ratios and Cysteine Residues on the Kinetic Stability and Unfolding Rates of Human Mitochondrial VDAC-2

**DOI:** 10.1371/journal.pone.0087701

**Published:** 2014-01-29

**Authors:** Svetlana Rajkumar Maurya, Radhakrishnan Mahalakshmi

**Affiliations:** Molecular Biophysics Laboratory, Department of Biological Sciences, Indian Institute of Science Education and Research, Bhopal, Madhya Pradesh, India; Russian Academy of Sciences, Institute for Biological Instrumentation, Russian Federation

## Abstract

Delineating the kinetic and thermodynamic factors which contribute to the stability of transmembrane β-barrels is critical to gain an in-depth understanding of membrane protein behavior. Human mitochondrial voltage-dependent anion channel isoform 2 (hVDAC-2), one of the key anti-apoptotic eukaryotic β-barrel proteins, is of paramount importance, owing to its indispensable role in cell survival. We demonstrate here that the stability of hVDAC-2 bears a strong kinetic contribution that is dependent on the absolute micellar concentration used for barrel folding. The refolding efficiency and ensuing stability is sensitive to the lipid-to-protein (LPR) ratio, and displays a non-linear relationship, with both low and high micellar amounts being detrimental to hVDAC-2 structure. Unfolding and aggregation process are sequential events and show strong temperature dependence. We demonstrate that an optimal lipid-to-protein ratio of 2600∶1 – 13000∶1 offers the highest protection against thermal denaturation. Activation energies derived only for lower LPRs are ∼17 kcal mol^−1^ for full-length hVDAC-2 and ∼23 kcal mol^−1^ for the Cys-less mutant, suggesting that the nine cysteine residues of hVDAC-2 impart additional malleability to the barrel scaffold. Our studies reveal that cysteine residues play a key role in the kinetic stability of the protein, determine barrel rigidity and thereby give rise to strong micellar association of hVDAC-2. Non-linearity of the Arrhenius plot at high LPRs coupled with observation of protein aggregation upon thermal denaturation indicates that contributions from both kinetic and thermodynamic components stabilize the 19-stranded β-barrel. Lipid-protein interaction and the linked kinetic contribution to free energy of the folded protein are together expected to play a key role in hVDAC-2 recycling and the functional switch at the onset of apoptosis.

## Introduction

The stability of a folded protein is largely a consequence of either thermodynamic or kinetic factors [1]–[5]. The vast majority of well-characterized soluble proteins and a few membrane proteins exhibit thermodynamic stability, wherein the equilibrium between the folded (N) and unfolded (U) protein forms is inclined in favor of the folded protein, due to the large negative equilibrium free energy of this state. Estimates of the change in unfolding free energy for such protein systems under thermal, mechanical or chemical stress can readily be determined *in vitro* [6]–[12] and *in vivo* [13], if they concur with the equilibrium unfolding model N↔U. This model assumes that the unfolding and refolding pathways, for a given condition, are superposable [1], [8], [14]. The occurrence of irreversibility, particularly in thermal denaturation experiments, leading to protein aggregation, suggests the existence of a kinetic barrier to the (un)folding process (N↔U→D), and in turn reflects kinetic stabilization of the refolded protein [3], [15].

The notion that membrane proteins, in particular, can exist in a kinetically stable state, rather than possessing thermodynamic stability, has gained popularity over the past few years [3]–[5], [16]. Unfolding of such proteins can proceed only with sufficient lowering of the large energy barrier separating the native form from the unfolded state(s). As the ‘folding equilibrium’ is no longer an exergonic (and thereby spontaneous) process, irreversibility and/or hysteresis ensue(s). Furthermore, the necessary existence of reversible equilibrium between protein (folded) ↔ protein (unfolded), protein-lipid and empty lipid moieties adds greater complexity to thermodynamic studies of transmembrane (TM) proteins [5].

Another increasingly accepted feature of membrane proteins is the observed dependence of protein stability on the lipid composition and concentration, with an increase in lipid/detergent concomitantly increasing stability [4], [17], [18]. Several studies have previously assessed the effect of lipid concentration and lipid-to-protein ratio (LPR) on the folding pathway(s) of TM β-barrels [19]–[21]. It has been reported that refolding of this class of proteins is nucleated only in the presence of a critical lipid or detergent concentration, which also determines whether hysteresis occurs in the folding and unfolding pathways. A minimum LPR, coupled with specific lipid or detergent preferences, is required to confer the maximum thermodynamic and kinetic stability to the refolded membrane protein.

Very few studies have extended the findings, directed towards thermodynamic and kinetic factors stabilizing bacterial outer membrane proteins, to eukaryotic membrane proteins [22], [23]. Studies that address the consequence of variation in LPR on the unfolding of transmembrane barrels *in vitro* are also available only for a few transmembrane proteins [19]–[21]. Analogous to the refolding studies, understanding membrane protein unfolding and factors that result in this process is also important, as it throws light on recycling of these proteins within cells. Furthermore, it allows an assessment of how the free energy barrier is overcome when such kinetically stabilized proteins are unfolded.

In this report, we have addressed the unfolding response of the human voltage-dependent anion channel isoform 2 (hVDAC-2) to a variation in the concentrations of lauryldimethylamine oxide (LDAO) and the contribution of thermodynamic *versus* kinetic components towards this stability. hVDAC-2, one of three isoforms seen in mammals, belongs to a family of highly conserved 19-stranded TM β-barrels of the mitochondrial outer membrane. The primary functions of VDACs are ion transport [24] and regulation of metabolite conductance (ATP, NADH etc.) [25]. Additionally, these barrels play a critical role in hexokinase regulation [26], cytochrome c release and mitochondria-mediated apoptosis [27].

**Figure 1:**
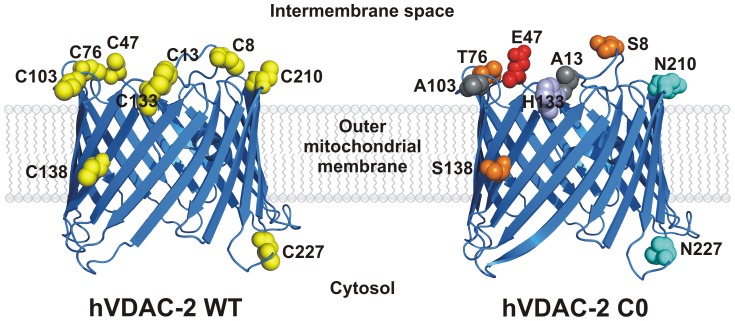
Cartoon representations of hVDAC-2 WT (left) and C0 (right). The structures were modeled using I-TASSER [55] using the crystal (2JK4 [29]) and NMR (2K4T [56]) structure of hVDAC-1 as the template. The cysteine residues have been highlighted as yellow spheres for the WT protein and they are largely oriented towards the intermembrane space in the 19-stranded model [22]. The corresponding mutated residues in C0 have been represented as spheres and colored according to the chemical characteristics of the amino acid.

While all three isoforms share significant sequence identity (>70%), hVDAC-1 and hVDAC-2 display antagonistic functions during apoptosis, with the latter being anti-apoptotic [28]–[30]. We have previously demonstrated that unlike hVDAC-1, hVDAC-2 refolds rapidly in LDAO micelles, remains stable for days and exhibits high thermal stability; however barrel stability is lowered in lipid vesicles [22]. While its structure can be modeled using hVDAC-1 [22], [30], when we consider the glaring functional differences between the two proteins, it becomes apparent that this could arise from subtle variations in the biophysical and biochemical properties dictated by the sequence variations between them. Cysteine residues have been shown to have no functional role in hVDAC-1 [31], [32]; hence the high number of cysteines in isoform 2 of VDAC (Figure S1) [22], an isoform restricted to mammals [28], is particularly interesting. Seven of the nine cysteine residues face the intermembrane space in the homology modeled structure of hVDAC-2 (Figure 1), and are strategically positioned to neutralize the reactive oxygen species (ROS) generated therein [30], [31], [33]. We therefore addressed the biophysical features of hVDAC-2 and compared it with a Cys-less mutant, in LDAO micelles [22], as we had previously established that cysteine residues contribute to barrel stability. We demonstrate that hVDAC-2 is stabilized by both thermodynamic and kinetic components and shows a non-linear LDAO dependence.

## Materials and Methods

### hVDAC-2 preparation

Human VDAC-2 gene was codon optimized, cloned in pET-3b without the signal sequence, and over-expressed in *E. coli* BL21(DE3) cells in the form of inclusion bodies, as reported earlier [22]. The Cys-less mutant (C0) was generated using multiple rounds of site-directed mutagenesis and generated using protocols similar to the wild type protein. hVDAC-2, purified from inclusion bodies using anion exchange chromatography, was lyophilized to obtain a white powder.

Refolding of both hVDAC-2 WT (hVDAC-2 wild-type) and C0 in 65 mM LDAO were carried out as described previously [22]. Briefly, denatured protein in 6 M guanidine hydrochloride (GdnHCl) was rapidly diluted 10-fold into the refolding mixture comprising 65 mM LDAO, 100 mM NaCl, 10 mM DTT (for WT only) prepared in 50 mM sodium phosphate buffer pH 7.2 at 4°C. The reaction was incubated for at 4°C for 4–6 h, dialysed against 100 mM NaCl and 50 mM phosphate buffer pH 7.2 (10 mM DTT for WT) and aggregated protein was removed by extensive centrifugation. The final refolding reaction used for all experiments contained 25 µM protein (0.8 µg/µL) in 65 mM LDAO, 50 mM sodium phosphate buffer pH 7.2, 100 mM NaCl and 10 mM DTT (in case of WT). This sample served as the stock protein for all experiments described herein (unless stated otherwise) and was diluted five-fold in various LDAO concentrations to achieve LPRs between 2600∶1–20000∶1.

In addition to the above denatured protein in 6 M GdnHCl was diluted either 50-fold (LPR 1000∶1 containing 5 µM protein, 5D) or 130-fold (LPR 2600∶1 containing 2 µM protein, 2D) in 50 mM sodium phosphate buffer pH 7.2, 100 mM NaCl and 10 mM DTT (for WT) containing 5 mM LDAO. These samples were together categorized as directly refolded, and served as controls. They primarily differed from the samples diluted from the refolding stock, in terms of the LPRs achieved in the refolding reaction, the presence of trace amounts of GdnHCl in them, which was not removed by dialysis and presence of aggregated protein as they were not subjected to centrifugation.

### Thermal denaturation experiments using circular dichroism

All CD measurements were carried out using the JASCO J-815 (JASCO Inc., Japan) CD spectropolarimeter equipped with a water-cooled Peltier module. Wavelength scans were obtained as described earlier [22]. Thermal denaturation measurements, which monitor temperature dependence (T-scans, [34]) of the far-UV circular dichroism (CD) spectrum was monitored at 215 nm from 4–95°C using a ramp rate of 1°C/min. All experiments were repeated at least three times using independent samples and checked for data reproducibility. Data were processed using minor modifications of reported methods [22], as described below.

Kinetics of unfolding in various pre-set temperatures (T) (isothermal unfolding kinetics, [34]) was studied by incubating the sample at defined temperatures between 65–95°C with a step size of 5°C, and data was acquired every 1 s at 215 nm. The experiment dead time was ∼30 s and could not be avoided. Data at 215 nm were converted to molar ellipticity (ME_215_) as reported earlier [35], normalized, and fitted to a single or double exponential function to derive unfolding rates, as described in the data analysis section.

### Thermal denaturation experiments using fluorescence anisotropy

Intrinsic indole fluorescence of the four Trp residues of hVDAC-2 were used to monitor the change in anisotropy as a function of temperature ranging from 65–80°C. All experiments were carried out on a FluoroMax-4 spectrofluorometer (Horiba Jobin-Yvon, France) equipped with an air-cooled peltier as described earlier [36]. Briefly λ_ex_ of 295 nm and λ_em_ of 340 nm were used, with excitation and the emission slit widths of 5 nm and an integration time of 5 s. Data acquisition was carried out every ∼16 s.

### Data analysis

Complete data processing and analyses were carried out using SigmaPlot v11.0 (Systat Software, Inc.) and Origin 8.1 (OriginLab Corporation), and final plots were generated using SigmaPlot v11.0. All experiments were carried out at least in triplicate, using freshly refolded samples. Rates and *T_m_* (midpoint of transition) were derived using the equations described below. Datasets obtained from T-scans were fitted individually, and the parameters calculated were averaged, to obtain the mean and standard deviation. In the case of isothermal unfolding kinetics, fits to the mean normalized data were used to derive the unfolding rates. Figures illustrate representative data for various experiments; mean data, wherever presented, are shown with standard deviations.

### T-scans calculations

Unfolded fractions (*f_u_*) were calculated using the equation:
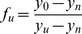



Here, *y_0_* is the observed molar ellipticity at a particular temperature; *y_n_* and *y_u_* are the molar ellipticities of the native and the unfolded proteins respectively. The *T_m_* was calculated using a two-state equation, as described earlier [37].

### Isothermal unfolding kinetics calculations

ME_215_ values obtained from isothermal unfolding kinetics were fit to either a single or double exponential decay function as described earlier [38], to derive the rates of unfolding. In cases where two transitions were obtained, the second transition, corresponding to protein aggregation, was analyzed. Here, data from the lower LDAO concentrations (5–65 mM) fit well to a double exponential decay and higher LDAO concentrations (80–100 mM) fit well to a single exponential decay function.

### Arrhenius plots and *E_act_* derivation

The natural logarithm values of the first rates (*k*_u1_) were linear between 5–30 mM LDAO, and hence were used to derive the activation energy for unfolding in these conditions. The activation energy (*E_act_*) was derived from fits of the Arrhenius plot to a linear function, using the following equation: slope  = −*E_act_*/R, where R is the gas constant (1.987 cal K^−1^ mol^−1^), as described earlier [38].

## Results and Discussion

### Human VDAC-2 isoform has nine cysteine residues that influence barrel-lipid interactions

The past three decades has seen substantial development in our understanding of VDAC structure and function. In humans, with the exception of liver, wherein lower levels of VDAC expression is observed, the three isoforms are nearly ubiquitous in all tissue types. The most abundant isoform 1 has two cysteine residues situated in the loop regions. It has been demonstrated earlier that these cysteines exist in the reduced state, are not necessary for barrel activity [31], and are not located at the predicted barrel dimerization interface [32]. hVDAC-2, on the other hand, possessed nine cysteine residues, which also exist in the reduced state in *in vitro* refolded protein [22]. It has been shown that the six cysteines of isoform 3 result in an additional ∼10% reduction in ROS-positive cells, when compared with cells over-expressing only VDAC-1 [39]. This suggests that the free thiol groups of hVDAC-3, and possibly hVDAC-2, could confer elevated protection levels from locally elevated ROS levels in the mitochondrial inter-membrane space.

In the modeled hVDAC-2 structure, the nine cysteine residues largely map to the loop segments of the 19-stranded β-barrel (Figure 1), possibly for ROS protection, suggesting that cysteines in these positions could be evolutionarily selected. Surprisingly, comparison of the sequences of the three hVDAC isoforms reveals a ‘conserved’ replacement of cysteines to carefully chosen residues that could have evolved owing to important contributions to the increased channel activity observed between eutherian VDAC-1, -2 and -3 (Figure S2) [40], [41]. Indeed, VDAC-1 is not only the most efficient porin [40], [41], but is also evolutionary the most recent channel isoform [30], [42]–[44], and it is tempting to speculate that key substitutions in hVDAC-3 and hVDAC-2 may have been selected for superior channel function. Hence, instead of substituting the cysteines with serines in hVDAC-2, we chose the corresponding residues from hVDAC-1 or hVDAC-3, so as to preserve possible functionality, and, in this process, obtain a mutant that resembles hVDAC-1.

As anticipated, substitution of these cysteines with the corresponding amino acid found in hVDAC-1 or hVDAC-3 results in negligible change in the modeled structure. However, we have previously demonstrated that mutation of the cysteine residues of hVDAC-2 results in altered biophysical properties of the barrel, stipulating an added structural role for cysteines in hVDAC-2, that is likely to be modulated by barrel-lipid interactions. Hence, we specifically probed the influence of LPR on the stability of hVDAC2 WT protein and its Cys-less mutant (C0). As shown in Figure 1, the Cys-less mutant has the following nine point mutations (substituted residues were derived from isoform 1 or 3) in the protein sequence: C8S/C13A/C47E/C76T/C103A/C133H/C138S/C210N/C227N. Of these, three substitutions (to Ser or Thr) are anticipated to largely retain the local polar contacts while four substitutions (to Glu, His or Asn) are likely to be more dramatic in changing the local polarity. However, considering that these substitutions are localized to the loop regions that stay solvent-exposed, it is presumed that these mutations are well accommodated.

### Barrel stability upon thermal denaturation is determined by LPR, but the effect is non-linear on both WT and C0

We addressed the stability of both the WT and Cys-less C0 mutant of hVDAC-2 in various LDAO concentrations by probing the effect of temperature as a denaturant, on protein behavior. Far-UV CD wavelength scans of both WT hVDAC-2 and the C0 mutants indicate comparable secondary structure content in both proteins, suggesting that a change in LPR does not adversely affect barrel formation, when the LPR is changed subsequent to folding. However, a marginal loss in secondary structure content is observed for samples directly refolded in low LPRs indicating that the absolute LDAO concentration is critical for the refolding process itself (data not shown). This observation is not surprising, considering that the presence of sufficient lipid or detergent is required for the proper refolding of membrane proteins [18], [21].

**Figure 2:**
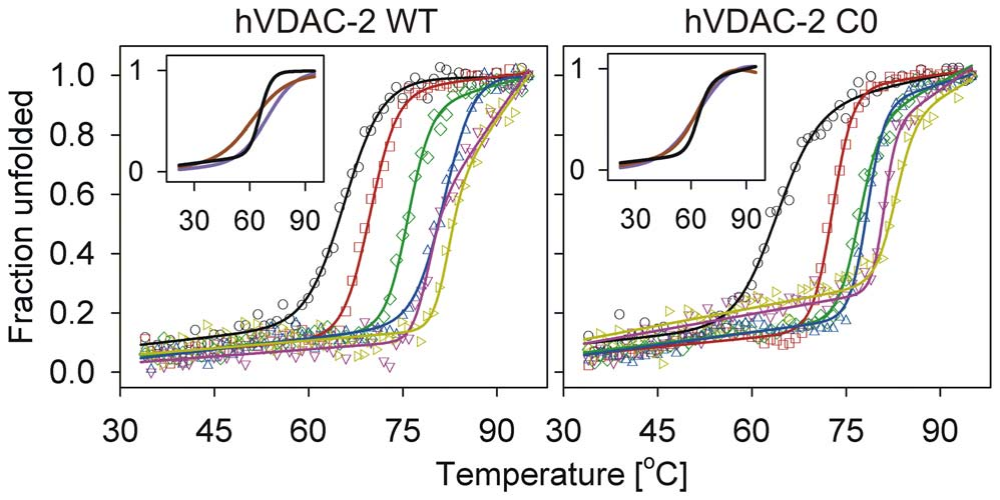
Thermal denaturation (T-scans) of refolded hVDAC-2 in various LDAO concentrations. Representative profiles of refolded hVDAC-2 WT (left) and C0 (right) in 5 mM (**○**, black), 13 mM (**□**, red), 30 mM (**⋄**, green), 65 mM (**▵**, blue), 80 mM (**▿**, dark pink) and 100 mM (**▹**, dark yellow) are shown here as unfolded fractions obtained at the various temperatures. The solid lines represent fits to a two-state equation. Note that hVDAC-2 C0 (right) shows substantial increase (∼30%) in the unfolded fraction by ∼80°C in 80 mM and 100 mM LDAO, even before commencement of the unfolding transition. Insets show the fits for samples directly refolded in 5 mM LDAO, to achieve an LPR of 2600∶1 (purple) or 1000∶1 (brown), and are compared with the 5 mM LDAO sample prepared by dilution (black) of the refolding stock to also achieve an LPR of 2600∶1 (see text for details). Actual data points have been omitted for clarity. The directly refolded samples, despite exhibiting a similar *T_m_*, show a distinct loss in unfolding cooperativity, when compared with samples prepared by dilution.

**Figure 3:**
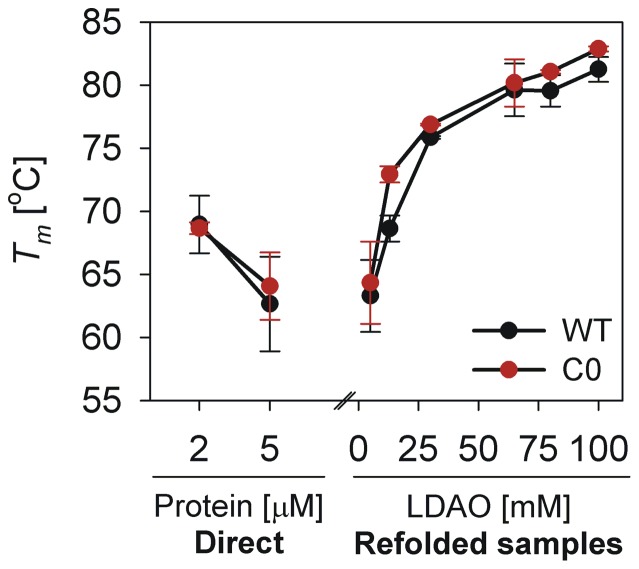
Correlation between *T_m_* of refolded hVDAC-2 and LDAO concentration. *T_m_* values obtained in the various LDAO concentrations described in this study show a near-exponential increase with LDAO concentration, till ∼65 mM. Increasing in LDAO concentration beyond ∼65 mM does not substantially change the *T_m_*. The *T_m_* of samples that were directly refolded in 5 mM LDAO (labeled ‘Direct’), has been included for comparison, to emphasize the importance of LPR as well as absolute LDAO concentrations during hVDAC-2 refolding. The latter samples exhibit a *T_m_* comparable to those prepared by dilution of the refolding stock to obtain the same LPRs; however, unfolding cooperativity is lost in the ‘Direct’ samples (see inset of Figure 2), emphasizing the importance of absolute LDAO concentration during refolding for protein stability. *T_m_* values were obtained from fits of three independent T-scan unfolding experiments to a two-state equation.

Membrane proteins also exhibit a strong dependence on lipid concentration, to remain stably refolded [18], [21]. Since the β-sheet content is largely analogous upon change in LPR in both WT and C0 proteins, we estimated barrel stability at various LDAO concentrations by comparing the thermal denaturation profiles from T-scan experiments. Both proteins undergo irreversible thermal denaturation, and show a transition from the native state (N) to the denatured/aggregated form (D) (N→D). We observe irreversibility even in high LDAO concentrations (LPR 20000∶1), although the magnitude of aggregation is lower, suggesting that excess LDAO does not necessarily confer greater stability to the barrel. LPR, however, plays an important role in determining the midpoint of this transition (*T_m_*) from N→D, in both proteins (Figure 2 and 3). Notably, the cooperativity of unfolding is retained in all cases, and the *T_m_* responds to an increase in LDAO amounts. A closer examination of the thermal transition of both WT and C0 samples prepared by ‘direct’ refolding in low LPRs (LPR 1000∶1 (D5) and 2600∶1 (D2)), however, points to a loss in cooperativity of the unfolding process, when compared with ‘refolded’ samples with similar LPRs, which were prepared by dilution of the refolding stock protein (Figure 2 inset). This is apparent from the broad transition range for the ‘direct’, spanning around ∼25°C, and ∼10°C for ‘refolded’ samples.

We determined the change in *T_m_* as a function of increasing LDAO, using the thermal transitions shown in Figure 2. Both WT and C0 exhibit an exponential increase in thermal stability, up to ∼30 mM LDAO; the change in the unfolding *T_m_* seen beyond 65 mM LDAO is negligible (Figure 3), suggesting that once an LPR of 13000∶1 is achieved, excess LDAO addition plays no significant role in hVDAC-2 thermal stability. Although marginal, we observe that the C0 barrel shows a persistently higher *T_m_* compared to the WT. We have previously observed that in 13 mM and 65 mM LDAO, the Stern-Volmer constants (which is a measure of the extent of solvent exposure of Trp residues) are greater for the C0 mutant, when compared with the WT protein [22]. This would suggest that the barrel-lipid interaction is weaker in the C0 mutant, which is contrary to our results from thermal denaturation measurements.

Interestingly, we observe stable baselines for thermal denaturation in high and low LPRs, until the unfolding event is nucleated. However, C0 shows a prominent (∼30%) loss in structure, particularly in high LPR, even before the unfolding transition commences at ∼80°C (Figure 2). Our previous results have pointed to a poorer association of the C0 mutant to LDAO [22]. Considering the marginally greater thermal stability displayed by C0, we are tempted to speculate that the weak(er) protein-lipid interactions in this protein are likely to be compensated by structural stabilization within the barrel. It is likely that C0 undergoes further destabilization with temperature in 80–100 mM LDAO, due to the presence of excess detergent in the system.

To summarize, our data provides us with three interesting observations on hVDAC-2 behavior. (i) The absolute LDAO concentration plays a critical role during barrel refolding and is vital for subsequent thermal stability. The latter is evident when we compare the *T_m_* of the 5 mM and 13 mM ‘refolded’ samples, both of which have an LPR of 2600∶1 (Figure 2). The 13 mM sample exhibits a *T_m_* of ∼70°C, whereas the 5 mM sample is reduced by 5–6°C, in both WT and C0. Furthermore, C0 unfolding is less cooperative than WT in 5 mM LDAO ‘refolded’ samples. (ii) Once refolded, subsequent LDAO dilution in both WT and C0 does not substantially perturb the barrel scaffold, suggesting that the refolded protein retains a detergent layer sufficient to preserve its folded state. However, thermal stability is lost in low LPRs. (iii) Optimal refolding and barrel stability is achieved by ∼65 mM LDAO and further detergent addition causes marginal destabilization of C0. Hence, an LPR of 2600∶1 is sufficient to drive hVDAC-2 refolding and maintain the 19-stranded barrel in a stably folded form.

### Isothermal unfolding kinetics indicate the presence of an irreversible unfolding intermediate

Proteins that undergo irreversible denaturation largely exhibit a transition from the native state to the aggregated state through an unfolded intermediate (N↔U→D) [3], [34]. Observation of the U state depends on the rate of U→D conversion; in most proteins, this rate is rapid, resulting in a pseudo two-state unfolding pathway from N→D [3]. While we are able to monitor completion of the denaturation process through T-scan experiments, the results do not allow for a demarcation between a three-state and a two-state process, and the possible occurrence of the unfolded intermediate in hVDAC-2. It has previously been argued that isothermal unfolding kinetics allows derivation of both the thermodynamics and kinetics of such unfolding processes [34], allowing the capture of an intermediate, if present, in thermal unfolding. We therefore examined the unfolding pathway(s) of hVDAC-2 WT and C0 using isothermal kinetics.

**Figure 4:**
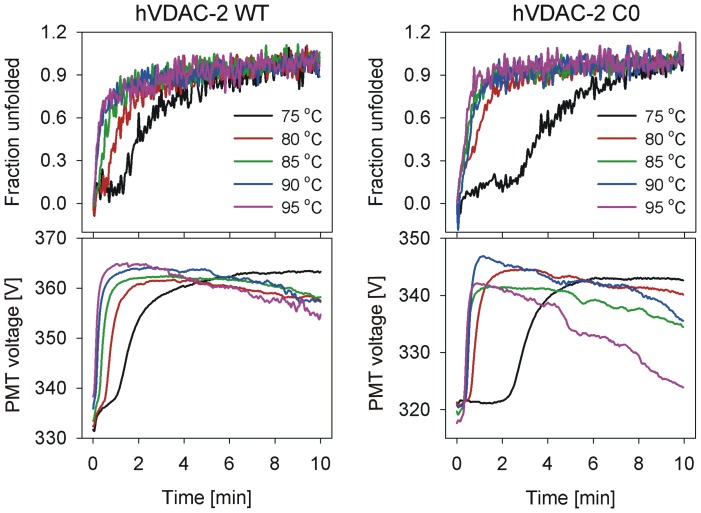
Isothermal unfolding kinetics monitored using ME_215_ for hVDAC-2. The upper panels show the representative data obtained in 30_215_ for both WT (left) and C0 (right). The lower panels show the corresponding change in the photomultiplier tube (PMT) voltage, which measures light scattering from the sample (color codes are the same for all the panels). Note the rise in PMT voltage as the protein loses secondary structure and aggregates, and the drop when the aggregates start to settle. The unfolding process starts a little later for C0 (for instance, compare the 75°C fraction unfolded data shown in black) although aggregation rate is faster for C0 (compare the 95°C PMT voltage data shown in purple).

Using CD ME_215_ values, we estimated the unfolding and aggregation rates of hVDAC-2 WT and C0, by monitoring changes in the β-sheet content under various isothermal conditions (T) and LDAO concentrations. We observe rapid protein unfolding, coupled with aggregation, within minutes of thermal denaturation (Figure 4). Interestingly, aggregation rates show a strong exponential component in C0 than WT, particularly at higher T, despite the absence of cysteine residues. This phenomenon is evident when we monitor the photomultiplier tube voltage with time at various T (Figure 4, lower panel). The initial rise (0–2 min) in the PMT value is due to the onset of aggregation. However, subsequent reduction as the unfolding process is completed is due to settling of the ordered aggregates, reducing the light scattering in the beam path, at 215 nm. Observation of differences in PMT values for both proteins suggests either the higher capacity of the C0 construct to self-associate or low aggregation tendency of WT, owing to its ability to interact better with LDAO.

**Figure 5:**
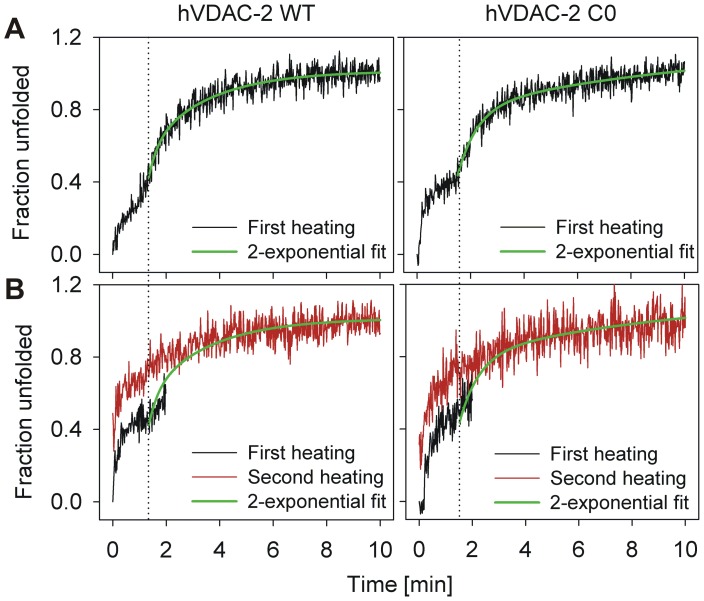
hVDAC-2 unfolding is irreversible, despite the presence of an unfolding intermediate. (A) 5 µM of refolded protein in 65 mM LDAO was subjected to thermal denaturation at 80°C, and the loss in ME_215_ (shown here after conversion to fraction unfolded) was recorded. Two transitions were seen in the case of both WT and C0, with the second transition starting at ∼2 min. (B) To check whether the first transition was due to reversible or irreversible unfolding, a fresh batch of refolded protein in 65 mM LDAO was subjected to 80°C for 2 min (black traces), after which the cuvette was immediately transferred to 4°C for 10 min before re-heating at 80°C (red traces). Visible aggregation was not observed after the initial 2 min heating. The second (red) trace, however, did not show the two transitions expected for this experimental condition, and instead only exhibited a single transition corresponding to protein aggregation. This indicates that the first transition is likely to arise from an irreversible change in protein conformation that does not correspond to the aggregated species. Shown in green are the fits of the second transition seen in (A) to an exponential function. The same fits are also depicted in (B) to highlight the difference in the profile of the re-heated samples (red trace) in the first 2 min of the recording, as well as demonstrate that similar end-points are achieved in both experiments.

As is seen in the T-scan experiments, isothermal unfolding also results in irreversible protein unfolding at all the LPRs studied. At higher temperatures, when T>*T_m_*, we observe rapid unfolding coupled with protein aggregation within minutes. Interestingly, at lower temperatures, when T<*T_m_*, we observe the occurrence of two sequential exponential unfolding events (for example, see the 75°C data in Figure 4). The first event could presumably arise due to the barrel unfolding process (U), which is then quickly followed by the irreversible aggregation event (D). These unfolding curves could therefore be characterized by two transitions, corresponding to N→U and U→D, respectively. To check if the first process (N↔U) was reversible, we subjected refolded hVDAC-2 WT and C0 in various LPRs to isothermal denaturation at T<*T_m_* for 2–3 min, cooled, and re-heated the protein. Here, despite secondary structure content being salvaged after cooling, both WT and C0 show a direct transition from N→D upon re-heating, and the intermediate U is not observed in the second heating cycle (Figure 5).

Hence, for hVDAC-2, isothermal denaturation at T<*T_m_*, therefore adopts the N→N*→D mechanism, where N* denotes a highly destabilized intermediate (with similar ME_215_ as N), which does not refold to the native structure and directly aggregates on re-heating. Notably, both transitions are irreversible, unlike previous observations for soluble proteins, which show a reversible unfolding but irreversible aggregation event [3], [45]. This observation seems to suggest that a part of the unfolding process is a kinetically controlled event [34].

### Unfolding and aggregation are sequential and irreversible at denaturation temperatures lower than *T_m_*

**Figure 6:**
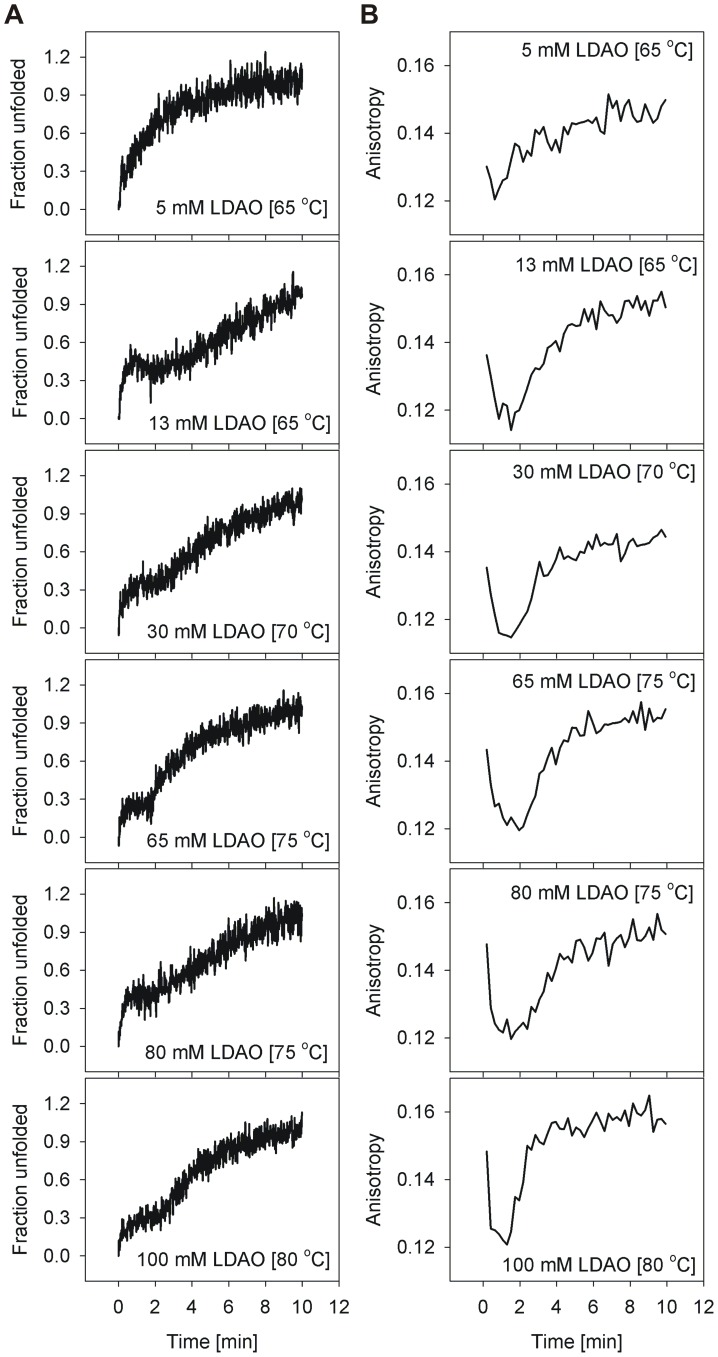
Comparison of the loss in ME_215_ and change in tryptophan anisotropy in isothermal unfolding kinetics of hVDAC-2 WT. Representative graphs illustrating (A) ME_215_ (converted to fraction unfolded) and corresponding Trp fluorescence anisotropy values (B) for all temperatures that gave rise to two transitions in the CD experiments. The change in tryptophan anisotropy was also monitored at these temperatures to understand the origin of the sequential unfolding events. The first CD transition is accompanied by a sharp decrease in the observed anisotropy, which we attribute to the initial unfolding event. ME_215_ recordings that showed only a single transition lack this initial decrease in anisotropy (not shown). In most of the LPRs and recording temperatures, the rise in sample anisotropy correlates well with the second transition, although the event appears to be faster in case of anisotropy measurements. This is likely to arise from the aggregation event being faster than the rate of loss of secondary structure content.

**Figure 7:**
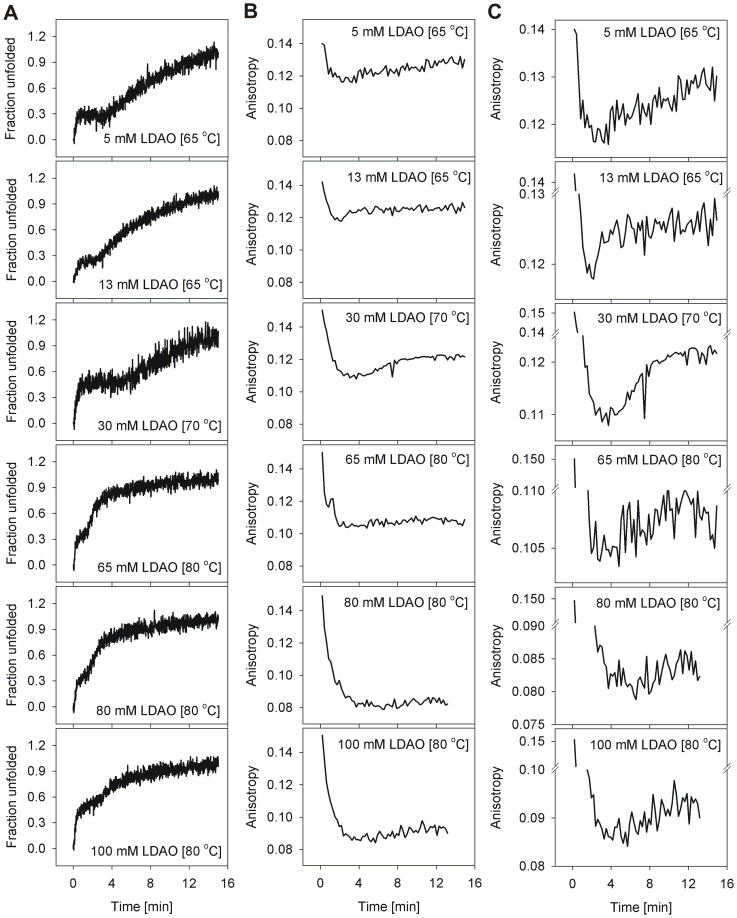
Isothermal unfolding kinetics of hVDAC-2 C0 monitored by comparing loss in ME_215_ with change in anisotropy. Shown are representative graphs illustrating the (A) loss in ME_215_ (converted to fraction unfolded) and (B and C) Trp fluorescence anisotropy. The temperatures at which two transitions were obtained in CD experiment for the different LDAO concentrations were used to monitor the change in tryptophan anisotropy, to understand the origin of the two transitions of C0. As seen in the case of WT, the first CD transition provides a concomitant rapid decrease in anisotropy. However, unlike WT, anisotropy values of aggregated C0 are lower. Furthermore, anisotropy of C0 decreases with increase in temperature (established by independent experiments, data not shown). The increase in anisotropy with time, corresponding to the second ME_215_ transition, although present, is therefore not as prominent in C0 as its WT counterpart. This second transition is therefore highlighted separately in (C). We speculate that this difference in anisotropy values arises from the varied affinity of both WT and C0 to LDAO. While aggregation is faster in C0 (see Figure 4), the corresponding change in anisotropy is marginal, since this construct has little affinity for LDAO molecules. Unfolded WT protein is presumably bound to sufficient LDAO that results in a greater change in anisotropy from 0.12 to 0.16.

The overall unfolding rate is linearly proportional to the unfolding temperature T, in both the barrels, especially when T<*T_m_* (discussed later). As noted above, at T>*T_m_*, barrel unfolding is characterized by an exponential loss in ME_215_, and follows an apparent two-step N→D mechanism, suggesting that unfolding and aggregation occur simultaneously. Therefore, in order to delink the unfolding and aggregation events in various LDAO concentrations, we monitored the isothermal unfolding kinetics at T<*T_m_*. Furthermore, to characterize these species, we examined the hVDAC-2 unfolding process using Trp fluorescence anisotropy, and compared the observed changes with the corresponding unfolding rates monitored using ME_215_ for WT (Figure 6) and C0 (Figure 7).

Refolded hVDAC-2 WT and C0 barrels exhibit an anisotropy in the range 0.13–0.15, which is lowered to 0.06–0.08 for the unfolded protein. In the unfolding kinetics, when T<*T_m_*, we observe a dramatic reduction in anisotropy values in the first 2–3 min (Figures 6B, 7B and 7C), which parallels the first transition of ME_215_ (Figures 6A and 7A). Protein unfolding, accompanied by dissociation of the barrel-LDAO structure, could result in the observed lowering of anisotropy. Subsequently, an increase in anisotropy, corresponding to the aggregation process, correlates well with the second transition of the CD unfolding curve. Our anisotropy measurements support a sequential unfolding (N→U) and aggregation (U→D) event at T<*T_m_*, and correlates with the two observed transitions from far-UV circular dichroism measurements.

Interestingly, the anisotropy of WT in isothermal unfolding kinetics increases to ∼0.16 from ∼0.12, with increasing LDAO (Figure 6B). However, in C0, the effect is reversed, with the anisotropy decreasing from ∼0.12 to ∼0.08, with increasing LDAO amounts (Figures 7B and 7C). The local environment of the four Trp residues in the soluble aggregates formed in both proteins, would determine the observed anisotropy values, with greater chromophore rigidity giving rise to higher anisotropy. Based on the opposing LDAO dependence of the anisotropy in WT and C0, we are tempted to speculate that the anomalous increase in WT anisotropy could arise from bound LDAO molecules that, despite structural collapse, are localized near the Trp residue(s). Such LDAO-bound protein, in turn, would undergo slower aggregation, as is seen for WT. C0, on the other hand, displays low LDAO affinity. Hence, the observed decrease in anisotropy of C0 correlates well with the formation of compact protein aggregates with exposed, conformationally unconstrained Trp residues, and accounts for its higher aggregation rate. The reason for this difference in affinity of WT and C0 to LDAO is presently unknown, and may perhaps stem from the cysteine residues in the WT sequence or the corresponding substitutions in C0. It is tempting to speculate that such differences in lipid affinity could also exist between isoforms 1 and 2 in the mitochondrial outer membrane, since hVDAC-1 has fewer cysteine residues.

### Unfolding rates and activation energy for both WT and C0 are distinct with the rates exhibiting LPR dependence

**Figure 8:**
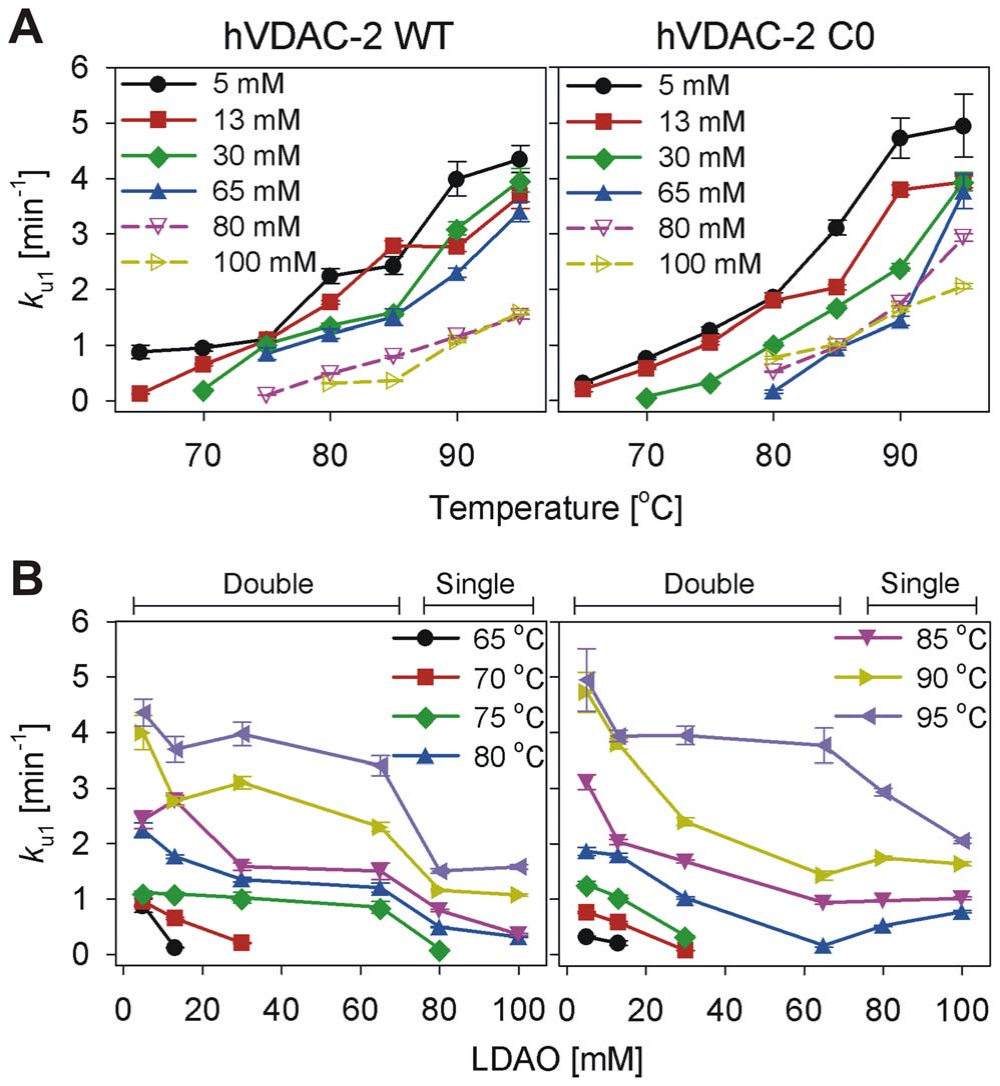
Comparison of the rate of unfolding of hVDAC-2 WT and C0. Rate of unfolding of refolded hVDAC-2 WT and C0 as a function of LDAO concentrations (A) and temperature (B) derived from isothermal kinetics experiments. Panel (A) highlights the decrease in unfolding rate with increasing LDAO concentration. Panel (B) highlights the increase in the unfolding rate with increase in temperature. Values for the solid symbols have been derived from the *k*_u1_ of the double exponential fits whereas the hollow symbols are for the single exponential fits. Figure legends in panel (B) are distributed in the left and right graphs. Error bars denote the goodness of the fit obtained by fitting the mean data of three independent experiments.

**Figure 9:**
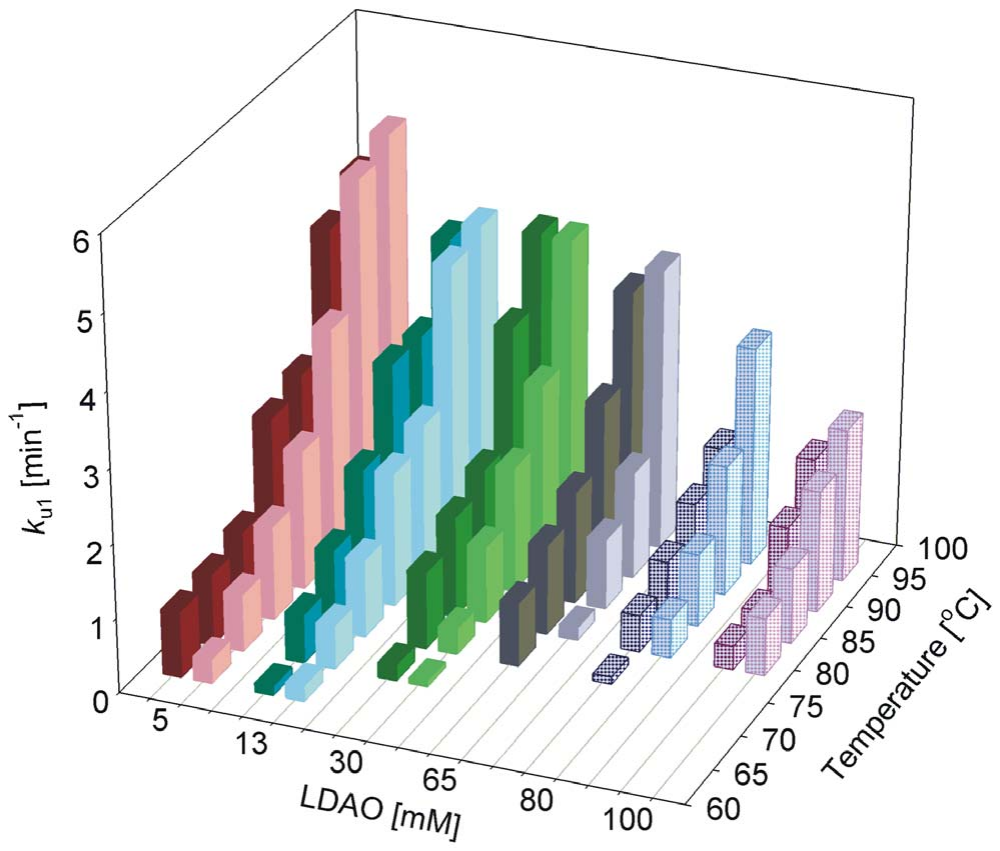
Unfolding and aggregation kinetics of hVDAC-2 at various LDAO concentrations. Refolded hVDAC-2 WT (left, dark colors) and C0 (right, light colors) in different LDAO concentrations, were subjected to denaturation at various temperatures, and change in ME_215_ was monitored using CD. Unfolding rates were derived by fitting the plots to either a double (solid bars) or single (dotted bars; 80 mM and 100 mM LDAO) exponential function. The fast rate (*k*_u1_) has been plotted against LDAO concentration and temperature to show its dependency to both of these parameters. Note how the overall *k*_u1_ values for C0 are lower between 13–65 mM LDAO. In 80–100 mM LDAO, C0 is less stable and undergoes a more rapid unfolding and aggregation, compared to WT. The rates have been derived by fitting the mean data of three independent experiments.

We fit the hVDAC-2 unfolding kinetics (U→D for T<*T_m_* or N→D for T>*T_m_*), obtained at various LPRs to either a single or a double exponential function, to derive protein aggregation rates (*k*_u1_, *k*_u2_ for the double exponential and *k*_u1_ for the single exponential fits). The results obtained are listed in Table 1 and compared in Figures 8 and 9. With increasing temperature, we observe an overall linear increase in the unfolding rate (Figure 8A), which is expected for processes such as protein unfolding, which are endergonic events. Comparison of the unfolding and aggregation rate (*k*_u1_) with increasing LDAO concentration also largely displays an overall linear change with LPR, with lower unfolding rates in higher LDAO concentrations (Figure 8B).

**Table 1:** Unfolding rate constants obtained from single or double exponential fits of isothermal kinetics experiments.

Temperature (T) [°C]	hVDAC-2 WT		hVDAC-2 C0	
	*k*_u1_ [min^−1^]	*k*_u2_ [min^−1^]	*k*_u1_ [min^−1^]	*k*_u2_ [min^−1^]
	**5 mM LDAO (LPR 2600∶1)** a			
**65**	0.882±0.12	-	0.322±0.01	-
**70**	0.953±0.06	-	0.761±0.02	-
**75**	1.113±0.03	-	1.261±0.06	-
**80**	2.242±0.13	0.340±0.11	1.863±0.08	0.176±0.10
**85**	2.434±0.16	0.345±0.13	3.112±0.13	0.346±0.07
**90**	3.997±0.31	0.675±0.11	4.729±0.36	0.821±0.11
**95**	4.354±0.25	0.338±0.05	4.952±0.56	0.738±0.09
	**13 mM LDAO (LPR 2600∶1)** a			
**65**	0.123±0.002	-	0.209±0.04	-
**70**	0.652±0.02	-	0.578±0.02	0.089±0.02
**75**	1.095±0.03	-	1.042±0.03	-
**80**	1.766±0.04	-	1.794±0.04	-
**85**	2.788±0.09	-	2.035±0.05	-
**90**	2.769±0.09	0.080±0.03	3.794±0.09	0.133±0.02
**95**	3.696±0.23	0.295±0.02	3.937±0.10	0.195±0.03
	**30 mM LDAO (LPR 6000∶1)** a			
**70**	0.206±0.01	-	0.075±0.002	-
**75**	1.027±0.10	-	0.339±0.005	-
**80**	1.352±0.04	-	1.023±0.02	-
**85**	1.586±0.06	-	1.681±0.03	-
**90**	3.104±0.11	0.161±0.03	2.398±0.07	0.222±0.11
**95**	3.972±0.21	0.459±0.04	3.947±0.17	0.537±0.09
	**65 mM LDAO (LPR 13000∶1)** a			
**75**	0.852±0.11	0.135±0.02	NO	NO
**80**	1.204±0.08	0.101±0.07	0.165±0.03	0.831±0.07
**85**	1.506±0.15	0.321±0.07	0.938±0.03	-
**90**	2.299±0.08	-	1.431±008	-
**95**	3.403±0.18	0.487±0.10	3.766±0.31	0.962±0.18
	**80 mM LDAO (LPR 16000∶1)** b			
**75**	0.099±0.003	NA	NO	NO
**80**	0.493±0.01	NA	0.520±0.01	NA
**85**	0.792±0.02	NA	0.974±0.02	NA
**90**	1.158±0.02	NA	1.744±0.04	NA
**95**	1.506±0.04	NA	2.933±0.07	NA
	**100 mM LDAO (LPR 20000∶1)** b			
**80**	0.316±0.008	NA	0.776±0.02	NA
**85**	0.361±0.02	NA	1.016±0.03	NA
**90**	1.072±0.02	NA	1.638±0.04	NA
**95**	1.582±0.04	NA	2.056±0.05	NA

a Values derived from fit to a double exponential function.

b Values derived from fit to a single exponential function; All error values represent goodness of fits. NA – Not applicable. NO – No denaturation observed.

A quick comparison of the unfolding rates between WT and C0 constructs suggests higher values in the case of C0. To examine this further, we compared the *k*_u1_ between both proteins and dependence of these values with temperature (T) and LDAO amounts. In 5 mM LDAO, the aggregation rate of C0 is marginally greater than WT (Figure 9). However, with increase in LDAO (between 13–65 mM), WT unfolding rates are faster at a given temperature, indicating that the C0 barrel is more stable in these conditions. The effect is reversed in 80–100 mM LDAO, wherein faster unfolding rates for C0 are observed. This reversal in WT and C0 behavior can be readily accounted for, when we consider the greater destabilization of C0 in high LDAO concentrations, and the concomitant loss in secondary structure from the T-scan experiments in 80–100 mM LDAO (Figure 2). Unfolding data obtained for both the constructs in high LPR fit well to a single exponent, indicating the absence of a rapid unfolding phase. We presume that this could merely result from ‘crowding’ by LDAO micelles, which effectively occludes protein-protein association, and thereby lowers aggregation rates. The observed variance in unfolding rates across the LDAO concentrations may point to altered kinetic stabilities of hVDAC-2 at these conditions, arising from changes in the native folded state of the *in vitro* refolded protein and/or the inability to reach the fully unfolded state within experimental timeframes.

**Figure 10:**
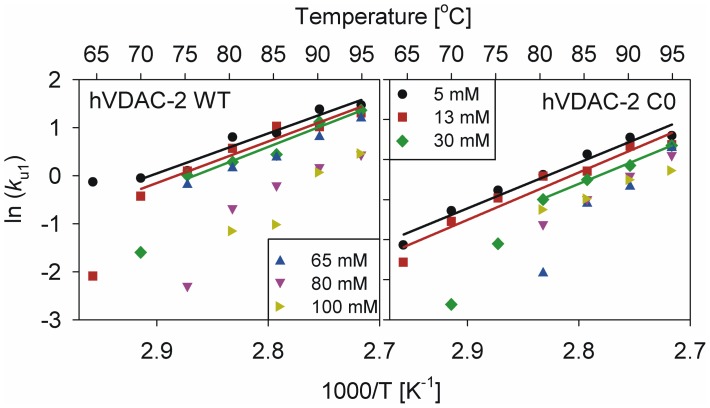
Arrhenius plots of the unfolding rate constants obtained from the isothermal unfolding kinetics using CD. Data for hVDAC-2 WT is shown to the left and C0 to the right. Fits to a linear equation could be obtained only in the lower LDAO concentrations (5–30 mM), and activation energy could therefore be derived for only these conditions. Also indicated along the upper axis is the absolute temperature used to measure the unfolding rates. Note that the figure legend is distributed across the two graphs.

Arrhenius plots, generated by comparing *k*_u1_ with T (Figure 10), show a linear dependency of the unfolding rates for both proteins in the lower LDAO concentrations, allowing for *E_act_* derivation only for these conditions. We obtained *E_act_* values of 16.54±1.85, 17.18±2.13 and 17.98±2.36 kcal mol^−1^ in 5, 13 and 30 mM LDAO, respectively, for the WT protein. In these LDAO concentrations, C0 provides *E_act_* values of 22.64±1.89, 23.53±2.62 and 22.77±1.06 kcal mol^−1^. Not surprisingly, elevated activation energies are seen for C0, in line with the observed marginal elevation in thermal stability of this protein from T-scan data (see Figure 3). Between 5–30 mM LDAO, *E_act_* is independent of detergent concentration for both proteins. In higher LDAO or when T<*T_m_*, non-linearity of the Arrhenius plot supports the existence of an unfolded intermediate [34]. We speculate that this is an on-pathway *un*folding intermediate that bridges the native and denatured states of the β-barrel, as reported earlier [34]. Further, our data indicate that the formation of this intermediate seals the fate of the barrel (Figure 5), signifying that this species is unlikely to exist also as an on-pathway *re*folding intermediate. We believe that differential stabilization of unfolding and refolding intermediates could give rise to the observed irreversibility of hVDAC-2 in the thermal denaturation process. Additionally, absence of a linear correlation of *k*_u1_ with temperature at high LPRs (65–100 mM LDAO) suggests the likely involvement of a thermodynamic component [34], in the stabilization of the 19-stranded barrel in high LDAO. Direct estimations of this thermodynamic contribution to the free energy of the folded protein are however convoluted by the prominent aggregation characteristic of the barrel, despite the presence of excess LDAO.

Comparison of *k*_u2_ derived from fits to a double exponential function shows an overall increase in rates with increase in temperature and/or lowering of LDAO concentrations (Table 1). However, a direct correlation to either parameter is not quantitative, precluding a definitive inference of the dependence of these rates to the aggregation event of hVDAC-2. Interestingly, our results are different from conclusions derived from similar experiments also carried out in LDAO micelles, on a bacterial reaction center [34], suggesting a protein-dependent variation of aggregation kinetics.

### hVDAC-2 stability is highest in intermittent LPRs and is lowered with increase or decrease in LDAO concentrations

**Figure 11:**
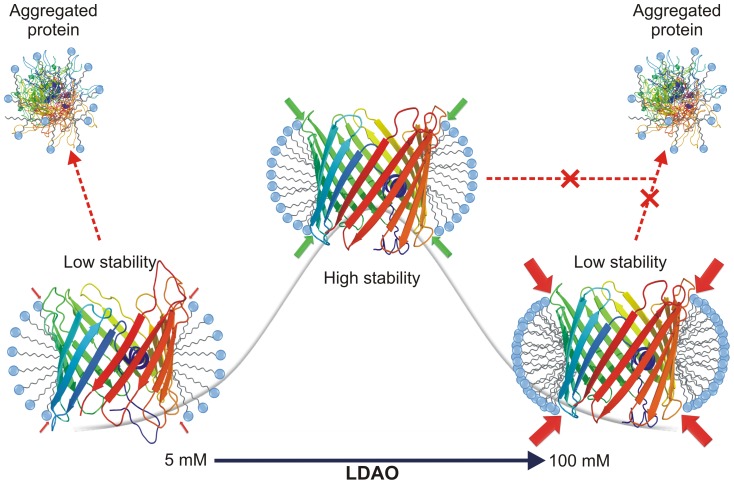
Schematic illustrating the relationship between hVDAC-2 stability and LDAO concentration. Highest barrel stability (represented by the gray line in the background) to thermal denaturation, is achieved only when hVDAC-2 is refolded from GdnHCl into an optimal LDAO concentration (65 mM) and LPR (2600∶1). Subsequent to folding, maintaining stability of the refolded WT and C0 barrels depends on the extent of lateral pressure (red or green filled arrows) exerted by the LDAO micelles on the protein scaffold. Optimal lateral pressure is achieved in LDAO concentrations of ∼13–65 mM (LPR 2600∶1–13000∶1; green arrows). Above and below this concentration, stability of hVDAC-2 is lowered due to the sub-optimal packing of LDAO against the barrel (indicated by red arrows). At low LDAO concentrations, the protein displays a tendency to aggregate (shown by dotted red arrow), while at higher concentrations aggregation is negligible. However, in high LDAO concentrations, very high lateral pressure of the micelles closely packed against the protein [46], may play a major role in barrel destabilization and the corresponding loss observed in the unfolding cooperativity.

Thermal denaturation experiments (T-scans) show protein stabilization beyond 65 mM LDAO, and an LPR of 13000∶1 (Figure 3). Further, isothermal unfolding kinetics suggest that for a particular value of T, the stability for both proteins (when we compare the unfolding rate at the various temperatures) is most favorable at ∼65 mM LDAO. Moreover, our experiments indicate that very high LDAO concentrations tend to destabilize the barrel structure. On the basis of these results, we summarize our observations on the response of hVDAC-2 to LDAO and the dependence of stability on LPR in Figure 11. In low LDAO concentrations (and corresponding low LPR amounts), hVDAC-2 can undergo refolding and attain the barrel scaffold under ambient conditions. However, the barrel is readily destabilized with marginal changes in temperature, indicating that the LPRs achieved are insufficient to retain the folded barrel state.

In high LDAO amounts, one would anticipate a proportionate increase in the stability of a membrane protein, including VDAC. Surprisingly, our studies reveal that hVDAC-2 exhibits a strong dependence on LPR for both folding [22] and unfolding kinetics (this study). It has been proposed earlier that lateral pressure exists in micelles when the detergent concentration is increased, which causes an increase in micelle size and packing. We are tempted to speculate that the ‘lateral pressure’ exerted by the increase in micelle size and compaction, with increase in LDAO concentration causes destabilization of hVDAC-2 in 80 and 100 mM LDAO. This phenomenon is further alleviated by the presence of salt (100 mM NaCl in our experiments), as it lowers electrostatic repulsion of the headgroups and promotes packing [46]. Hence, with LDAO as the refolding medium, both high and low LPRs cause hVDAC-2 destabilization, although the mechanism is different in both cases.

## Conclusion

Biophysical characterization of the 19-stranded hVDAC-2 β-barrel, which is indispensable for cell survival [28], can provide us with key insight into the factors influencing barrel folding and stability. In this study, we have specifically detailed the effect of LPR on hVDAC-2 barrel behavior. Studies on bacterial TM β-barrels have demonstrated that a change in the lipid concentration influences the folding kinetics [21], [47]; however, this has little bearing on barrel unfolding rates. In hVDAC-2, we observe a surprising dependence on the absolute LDAO concentration as well as LPR, on heat-mediated protein unfolding. Additionally, in thermal denaturation of hVDAC-2, the unfolded intermediate observed at T<*T_m_* does not recover to the native barrel structure, unlike the Lumry-Eyring N↔U→D model that is usually followed by kinetically trapped proteins [3], implying a dual contribution of both thermodynamic and kinetic components in mediating hVDAC-2 ↔ LDAO interaction. While the *in vivo* stabilization of hVDAC-2 may also arise primarily from the kinetic component, when we consider how membrane proteins are largely dependent on the surrounding lipid environment for structural integrity, it is possible that a thermodynamic component is also involved in hVDAC-2 stability [5], [34]. Alternatively, it can be argued that there exists a metastable unfolding intermediate which can no longer restore the native protein conformation, since it is not present in the folding pathway, allowing for a modification of the Lumry-Eyring model to N→N*→D at T<*T_m_* and N→D at T>*T_m_*, for hVDAC-2.

The subtle yet significant behavioral differences observed upon mutation of the cysteine residues of hVDAC-2 WT are also surprising, particularly when we consider that the substitutions were generated based on the corresponding residues observed in isoforms 1 or 3, in place of cysteines of hVDAC-2. Since temperature targets intra-protein non-covalent interactions, the marginally greater thermal stability of C0 reflects a better structured barrel, in line with our previous observation [22]. However, our results demonstrate that this structural advantage comes at the cost of lowered protein-lipid interactions, particularly at high LDAO. Lateral pressure generated on the folded protein by densely packed micelles in higher LPRs possibly mimic that of lipid bilayers, and cause hVDAC-2 destabilization, resulting in the formation of a kinetically trapped metastable intermediate state of the barrel (Figure 11). Rapid protein unfolding in high LDAO therefore ensues upon thermal denaturation. Indeed, our previous observations on biophysical behavior of hVDAC-2 upon increasing phosphocholine (14-C) levels indicate a near-exponential loss in thermal stability [22] that likely arises from curvature constraints imposed by the rigid phosphocholine bilayer on the asymmetric β-barrel structure.

The effect of lipids on VDAC from various organisms has been studied to some extent [48], [49]; however, to our knowledge, the role of lipids in hVDAC-2 function and stability has not been well examined. Cardiolipin, in particular, has been shown to prevent oligomerization [49] and catalyze voltage asymmetry of *Neurospora crassa* VDAC [48]. In addition to this, the mitochondrial outer membrane has higher LPR in comparison to the inner membrane [50]. Hence, a change in the lipidic environment and the lateral pressure, coupled with differential expression and membrane insertion of Bcl-2 family of apoptotic proteins, during initial stages of apoptosis, may together influence the permeability of the mitochondrial outer membrane [48], [49], and thereby modulate the functioning of various VDAC isoforms during apoptosis and cytochrome c release [44]. It is interesting to conjecture that such changes in lipid composition and LPR could influence the lipid packing efficacy and interaction strength of hVDAC-2, in a manner similar to our observations in LDAO. Such changes in the biophysical properties of the barrel could adversely affect its BAK interaction efficiency and the associated anti-apoptotic function [28]. However, since the micellar system of LDAO is not necessarily a membrane mimetic of the outer mitochondrial membrane, our inferences are presently hypothesis-driven. Our hVDAC-2 refolding screens have, so far, been successful only with micellar systems, and the barrel stability is substantially lowered in phosphocholines [22]. On the contrary, hVDAC-1 displays the ability to reconstitute in several lipids (10-16C phosphocholines) and detergents [29], [51], [52], including LDAO [29] and DMPC [52], suggesting that subtle differences in sequence may give rise to the inherent behavioral differences between the two isoforms *in vitro*, and possibly, *in vivo*. If stably refolded forms of hVDAC-2 can be achieved *in vitro*, it would be of great biophysical interest to examine the behavioral properties of this barrel in various lipid bilayer systems.

Could the subtle structural differences and the altered lipid-protein interactions between hVDAC-2 WT and C0 be compared with the *in vivo* behavioral and functional differences between hVDAC-2 and hVDAC-1 (which is closer to our C0 construct in terms of sequence)? As hVDAC-1 is the primary transporter of metabolites across the membrane, it is likely that barrel stability and structural rigidity is critical for its function, whereas hVDAC-2 may have alternative roles such as maintaining intracellular ROS levels [53], [54], and as an anti-apoptotic agent [28]. A detailed analysis of hVDAC-2 structure and residue-wise mapping of key interaction sites of this barrel with both its lipid environment as well as anti- and pro-apoptotic agents is expected to throw new light on our understanding of mitochondria-mediated apoptosis.

## Supporting Information

Figure S1 **Multiple sequence alignment of VDACs found in different species.** Sequences in green belong to lower eukaryotes or plants, sequences in red and black refer to VDAC-1 and VDAC-2, respectively, of higher eukaryotes generated using Clustal Omega (https://www.ebi.ac.uk/Tools/msa/clustalo/). Only isoform 1 and 2 have been shown for clarity, and the cysteines have been highlighted in yellow. Note the abundance of cysteines in mammalian VDAC isoform 2.
(TIF)

Figure S2 **Multiple sequence alignment of hVDAC-1, 2 and 3, with cysteines highlighted in yellow.** The cysteines that have conserved mutations in either of two isoforms of hVDAC have been boxed.
(TIF)
