# Supplementary figures and images for: Influence of Protein – Micelle Ratios and Cysteine Residues on the Kinetic Stability and Unfolding Rates of Human Mitochondrial VDAC-2
Source: PLoS One. 2014 Jan 29;9(1):e87701. doi: 10.1371/journal.pone.0087701 (PMC3907894; doi:10.1371/journal.pone.0087701)

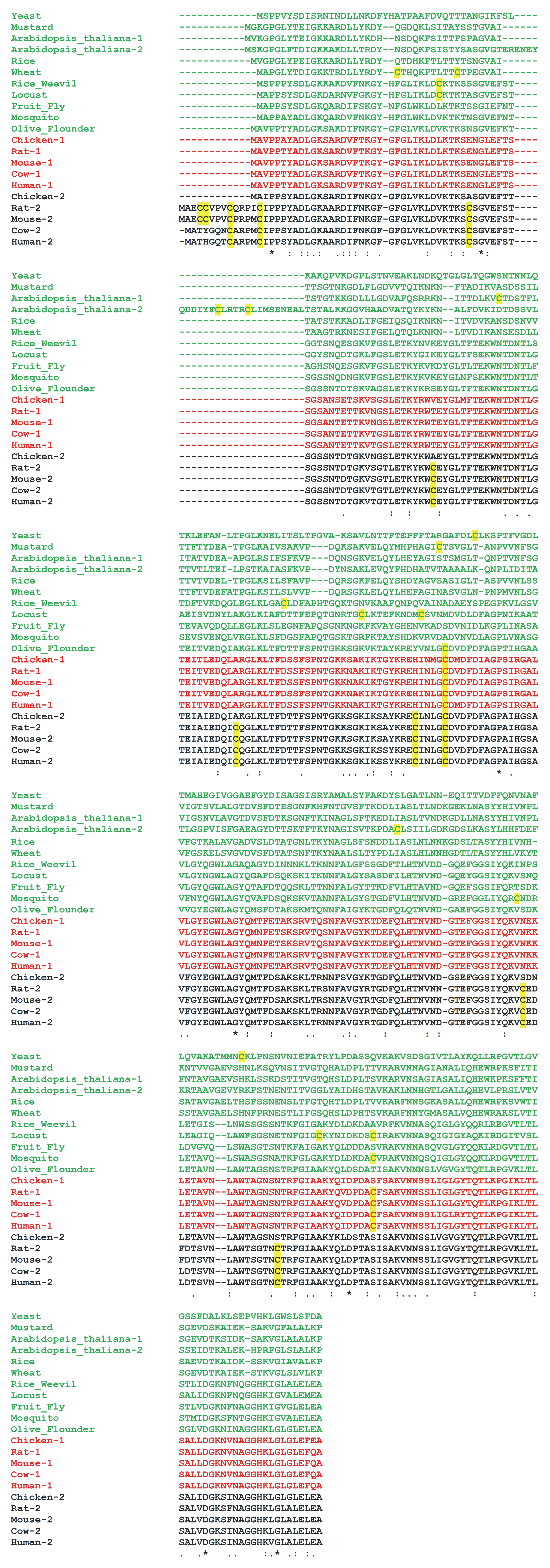

Supplement: Figure S1 — Multiple sequence alignment of VDACs found in different species. Sequences in green belong to lower eukaryotes or plants, sequences in red and black refer to VDAC-1 and VDAC-2, respectively, of higher eukaryotes generated using Clustal Omega (https://www.ebi.ac.uk/Tools/msa/clustalo/). Only isoform 1 and 2 have been shown for clarity, and the cysteines have been highlighted in yellow. Note the abundance of cysteines in mammalian VDAC isoform 2. (TIF) [file pone.0087701.s001.tif]

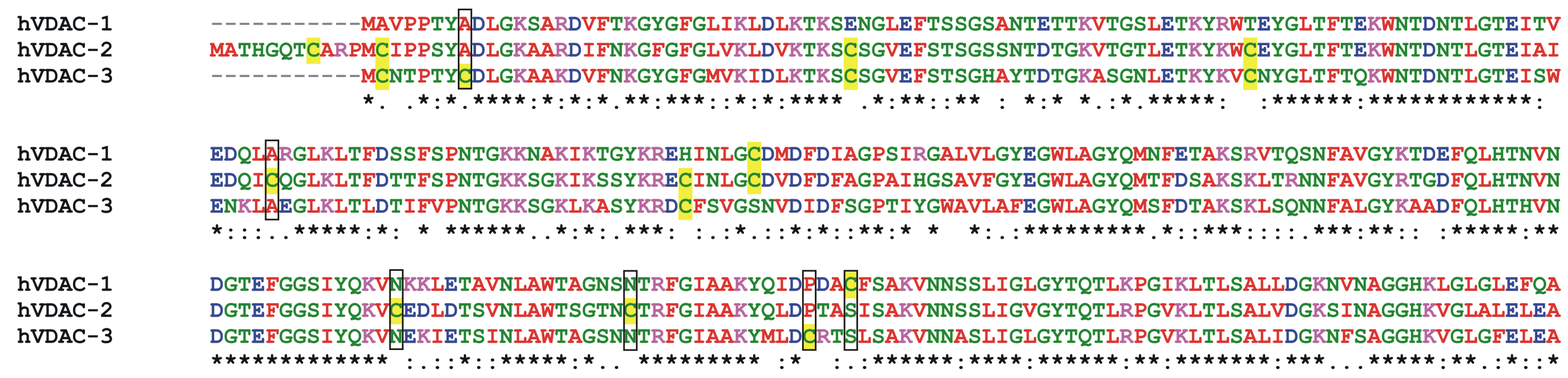

Supplement: Figure S2 — Multiple sequence alignment of hVDAC-1, 2 and 3, with cysteines highlighted in yellow. The cysteines that have conserved mutations in either of two isoforms of hVDAC have been boxed. (TIF) [file pone.0087701.s002.tif]
